# Machine learning models based on clinical indices and cardiotocographic features for discriminating asphyxia fetuses—Porto retrospective intrapartum study

**DOI:** 10.3389/fpubh.2023.1099263

**Published:** 2023-03-20

**Authors:** Maria Ribeiro, Inês Nunes, Luísa Castro, Cristina Costa-Santos, Teresa S. Henriques

**Affiliations:** ^1^Institute for Systems and Computer Engineering, Technology and Science (INESC-TEC), Porto, Portugal; ^2^Computer Science Department, Faculty of Sciences, University of Porto, Porto, Portugal; ^3^Institute of Biomedical Sciences Abel Salazar, University of Porto, Porto, Portugal; ^4^Centro Materno-Infantil do Norte—Centro Hospitalar e Universitário do Porto, Porto, Portugal; ^5^Centre for Health Technology and Services Research (CINTESIS), Faculty of Medicine University of Porto, Porto, Portugal; ^6^CINTESIS@RISE, MEDCIDS, Faculty of Medicine, University of Porto, Porto, Portugal; ^7^School of Health of Polytechnic of Porto, Porto, Portugal

**Keywords:** non-linear methods, neonatology, fetal heart rate, cardiotocography, perinatal asphyxia

## Abstract

**Introduction:**

Perinatal asphyxia is one of the most frequent causes of neonatal mortality, affecting approximately four million newborns worldwide each year and causing the death of one million individuals. One of the main reasons for these high incidences is the lack of consensual methods of early diagnosis for this pathology. Estimating risk-appropriate health care for mother and baby is essential for increasing the quality of the health care system. Thus, it is necessary to investigate models that improve the prediction of perinatal asphyxia. Access to the cardiotocographic signals (CTGs) in conjunction with various clinical parameters can be crucial for the development of a successful model.

**Objectives:**

This exploratory work aims to develop predictive models of perinatal asphyxia based on clinical parameters and fetal heart rate (fHR) indices.

**Methods:**

Single gestations data from a retrospective unicentric study from Centro Hospitalar e Universitário do Porto de São João (CHUSJ) between 2010 and 2018 was probed. The CTGs were acquired and analyzed by Omniview-SisPorto, estimating several fHR features. The clinical variables were obtained from the electronic clinical records stored by ObsCare. Entropy and compression characterized the complexity of the fHR time series. These variables' contribution to the prediction of asphyxia perinatal was probed by binary logistic regression (BLR) and Naive-Bayes (NB) models.

**Results:**

The data consisted of 517 cases, with 15 pathological cases. The asphyxia prediction models showed promising results, with an area under the receiver operator characteristic curve (AUC) >70%. In NB approaches, the best models combined clinical and SisPorto features. The best model was the univariate BLR with the variable compression ratio scale 2 (CR2) and an AUC of 94.93% [94.55; 95.31%].

**Conclusion:**

Both BLR and Bayesian models have advantages and disadvantages. The model with the best performance predicting perinatal asphyxia was the univariate BLR with the CR2 variable, demonstrating the importance of non-linear indices in perinatal asphyxia detection. Future studies should explore decision support systems to detect sepsis, including clinical and CTGs features (linear and non-linear).

## 1. Introduction

Perinatal asphyxia is characterized by an impaired exchange of respiratory gases (oxygen and carbon dioxide) between the mother and fetus, resulting in hypoxemia (decreased oxygen in the blood) and hypercapnia (increased carbon dioxide), accompanied by acidosis metabolism and tissue damage (1, 2).

The incidence rate of perinatal asphyxia in developed countries ranges from 1 to 5/6 cases per 1,000 live births (3). However, in developing countries, the incidence is higher, reaching 26.5 cases per 1,000 (2, 3). In addition, asphyxia is the third most common cause of neonatal death (23%), after preterm birth (28%), and serious infections (26%) (1, 4). One of the main reasons for these high incidences is the lack of consensual methods of early diagnosis for this pathology.

The causes of perinatal asphyxia can be maternal or fetal. There are several clinical risk factors for perinatal asphyxia, including maternal age, prolonged rupture of membranes, multiple births, non-attendance for prenatal care, low fetal weight, malpresentation, preeclampsia, increased labor with oxytocin, and abnormal fetal heart rate (fHR) (2, 5, 6). Asphyxia can still occur in utero during childbirth or in the immediate postnatal period, and in many cases, the timing of asphyxia cannot be established with certainty (2).

There has yet to be an agreement among the various authors regarding the diagnosis of asphyxia. However, the criteria of the American College of Obstetrics and Gynecology and the American Pediatric Association are based in pH, Apgar score, and the presence of neurological manifestations (7–9). Although, sometimes the diagnosis is difficult and proves to be late for many babies. Several biomarkers that identify kidney, central nervous system, or cardiac damage, to more specifically access the mechanism of hypoxia and be able to predict perinatal asphyxia, have been analyzed recently (2). Abiramalatha et al. (10) analyzed troponin-T concentrations in asphyxiated newborns and correlated concentrations with clinical outcomes. Patel et al. (11) used the uric acid/creatinine ratio as an additional marker for predicting perinatal asphyxia and compared it with blood gas analysis in monitoring the Apgar score. Mikkelsen et al. (12) studied how birth asphyxia, as measured by the pH of blood in the umbilical artery cord, is associated with attention deficit hyperactivity disorder in childhood.

The prediction of perinatal asphyxia is still a problem in the provision of health care. Understanding the probability of birth asphyxia risk is very important for better clinical care and medical intervention. A correct and easily obtained clinical prognosis is essential for predicting perinatal asphyxia. It is crucial to develop new bio and physiomarkers with greater specificity in the diagnosis and more accurate prediction models. However, several intrapartum events can cause asphyxia. It is vital to recognize a fetus presenting a pathological cardiotocographic signal (CTGs) at the delivery time, which may imply possible hypoxia and perinatal asphyxia (13–16). Therefore, the CTGs interpretation is essential. Besides the linear CTGs indices, entropy and compression are among the most used non-linear methods for predicting asphyxiation (17).

A frequent problem in medical data is the imbalance caused by the fact that the number of observations belonging to the two groups (asphyxia/non-asphyxia) is very different. An unbalanced dataset is a big challenge because failure to solve this problem can lead to classifiers being biased. Recently, da Silva Rocha et al. (18) reviewed the literature on computer models for mortality prediction, covering stillbirth, perinatal, neonatal, and infant deaths, and found that only 50% of studies addressed the crucial problem of the unbalanced dataset.

The main objective of this exploratory study is to develop predictive models that detect perinatal asphyxia using retrospective databases of CTGs in conjunction with clinical factors. For this purpose, we used clinical information and linear and non-linear CTG features to develop and compare perinatal asphyxia predictive models using logistic regression and Bayesian networks.

## 2. Data

In this study, we used data from a retrospective study that contained anonymized data from single gestations' childbirth from Centro Hospitalar e Universitário do Porto de São João (CHUSJ) between 2010 and 2018. Of the 22,648 cases that comprise the original dataset, 72 were diagnosed by the medical team with perinatal asphyxia (0.32%). In this study, the 22,524 subjects born alive were considered. The group with perinatal asphyxia, defined by the International Classification of Diseases, 9th Revision, Clinical Modification (ICD-9-CM), comprises 15 fetuses with a CTGs record in the last hour before birth.

For the non-asphyxia (suspicious) group, individuals with CTGs in the last hour before birth with a pH value record were considered (2,991 cases). From these cases, 502 cases were randomly selected and stratified by gestational age and gender.

The data was collected from two information systems: the Virtual Care's ObsCare system (19) and the Omniview-SisPorto (20). The ObsCare is an electronic clinical record system implemented in several Portuguese hospitals that support gynecology and obstetrics departments by storing data during pregnancy and birth. On the other hand, the Omniview-SisPorto acquires CTGs and analyzes them following the International Federation of Gynecology and Obstetrics guidelines (20, 21).

### 2.1. Clinical variables

The clinical variables obtained from ObsCare included in the database were organized into four groups: parturient, pregnancy, delivery, and fetus/newborn. (a) Parturient: age, body mass index (BMI), blood group (GS—A, AB, B, or O), RH system (RH positive or RH negative), and allergies. (b) Pregnancy: type of pregnancy (spontaneous or stimulation). (c) Labor: gestational age, fetal presentation (cephalic, non-cephalic), increased labor with oxytocin, intrapartum fever, and type of delivery (c-section, vaginal, vacuum). (d) Fetus: gender (male or female), and third-trimester ultrasound fetal weight percentile adapted to Portuguese reality (22).

### 2.2. SisPorto features

The traces from the last 60 min before delivery were analyzed using Omniview Sisporto 4.0 (20). The linear features considered were: fHR base and basal line, accelerations, fetal movement, number of contractions, average and abnormal short-term variability (STV), average and abnormal long-term variability (LTV), saltatory index, mild, intermediate, prolonged, and repetitive decelerations, and yellow, orange, red, and red without ST alerts. A full description of the indices can be found at (20, 23).

### 2.3. Non-linear indices

In this section, we describe the non-linear methods used to characterize the complexity of the fHR signals, for which we use the CTGs 60 min before birth.

Entropy and compression are two of the most applied non-linear measures to predict fetal pathologies. These measures have already been successfully used in asphyxia-related work (17).

A time series' entropy measures how irregularly distributed, or unpredictable, each new observation is. In other words, the entropy rate increases monotonically with the level of randomness (24). The concept of sample entropy (SampEn), introduced in 2000 by Richman and Moorman (25), is becoming the entropy most applied to heart rate time series (26). Furthermore, the multiscale entropy (MSE), proposed by Costa et al. (24, 27) has also been widely employed in biomedical signal analysis considering system information on different time scales.

The MSE method comprises two parts:

1. Construction of the time series scales: using the original signal, a scale (*s*) is created through a coarse-graining procedure by replacing *s* consecutive points with their average;
2. Computation of the entropy index for each time series scale. We apply SampEn with *m* = 2 and *r* = 0.15 × *standarddeviation* in this work.

The MSE curve is obtained by plotting the entropy value as a scale function. The entropy indices used were the entropy obtained from the original scale and scale 2 (SampEn1 and SampEn2, respectively) and the three indices related to the multiscale sample entropy computed as:

1. MSEsum—the sum of the entropy values of the first five scales;
2. MSEslope—the slope of the linear regression line best fitting the entropy values over on the first five scales;
3. MSEss—the product of MSEsum and MSEslope.

Another complementary approach is the Kolmogorov complexity one, which considers the amount of information in an object as the length of the object's smallest description. The Kolmogorov complexity attempts to answer how “random” an object is concerning the number of bits necessary to describe it. This measure is not computable but can be approximated using compressors. In this work, we used the bzip2 compressor (28).

We extended the MSE algorithm concept and combined it with the compression by replacing the second step, forming the multiscale compression (MSC). The compression indices used were the compressed file size for original scale and scale 2 (SC1 and SC2, respectively), and the three indices extracted from the multiscale compression curve (MSCsum, MSCslope, and MSCss) obtained analogously to the three indices of the MSE. Due to the order of magnitude the values of the MSCsum variable were divided by 10,000.

We also compute the compression ratio (CR) for each scale as follows:


(1) $$\begin{array}{l} CR=\frac{\text{size of compressed file for each scale}}{\text{size of file for each scale}}\times 100 \end{array}$$

The same five indices were considered, similarly to the previous indices (CR1, CR2, MSCsumCR, MSCslopeCR, and MSCssCR).

Therefore, 15 non-linear indices were computed (SampEn1, SampEn2, MSEsum, MSEslope, MSEss, SC1, SC2, MSCsum, MSCslope, MSCss, CR1, CR2, MSCsumCR, MSCslopeCR, and MSCssCR).

## 3. Prediction models

Data mining and machine learning have achieved significant results in various fields. Due to the success of these methods, many researchers have used machine learning algorithms in medical analyses (29). Prediction or classification models have great potential in predicting pathologies, identifying inefficiencies, improving clinical practices, and reducing costs.

In this work, we use binary logistic regression (BLR) and Bayesian network classifiers (Naive-Bayes) to analyze data and develop predictive models.

### 3.1. Binary logistic regression model

The binary logistic regression model is widely used in medical research and is part of a family of statistical models called generalized linear models. Recently, many researchers have applied this method to predict perinatal asphyxia (30–32).

The model produces odds ratio (ORs), which suggest an increase, decrease, or no change in the odds of being in an outcome category with an increase in predictor value (33). This work applies the univariable and multivariable BLR to predict perinatal asphyxia.

### 3.2. Bayesian model—Naive-Bayes

Traditional statistical methods, such as logistic regression, have difficulty dealing with large amounts of data. In recent years, new methods using machine learning techniques have emerged and their applications have increased. Due to its ability to provide causal inference, one of the most used machine learning techniques in medicine is Bayesian Network (34, 35).

Bayesian networks were introduced by Pearl (36) as a formalism to represent and reason with models of problems involving uncertainty, adopting probability theory as a basic framework (37). Initially, Bayesian networks were built manually, but due to the large amount and constant increase of data, there was a need to develop algorithms to learn networks from data. Learning Bayesian networks from data has two components: (1) the structure of the networks and (2) the parameters (conditional probability tables) (38). The Bayesian network has the advantages of dealing with missing data and modeling the interdependence between two variables even when the relationship between them is unknown (39).

There are many versions of Bayesian network classifiers (40, 41). In this work, we will apply one of the most popular classifiers—Naive Bayes (NB) classifier (42). NB classifier assumes the stochastic independence of the features given the class. It differs from other machine learning methods in that there is not an explicit search for a possible hypothesis but the product of the probabilities of each attribute (43). NB is a simple probabilistic classifier with fast techniques, robust to the presence of irrelevant attributes, and the dimension of its decision model is independent of the number of subjects.

## 4. Data balancing techniques

Unbalanced datasets are relevant and commonly observed in pathology detection problems that can significantly impact the classification performance of machine learning models. Several solutions have been proposed to deal with unbalanced datasets (44, 45) and the problem was solved by data resampling at the pre-processing data level. The basic idea of unbalance is to resample the original dataset, either by oversampling the smallest class or subsampling the largest class until the class sizes are approximately the same. In general, resampling techniques can be divided into three categories [undersampling, oversampling, and hybrid sampling (HS)].

Undersampling (US): the dataset is balanced by randomly excluding majority class instances. The main limitation of this method is that we can discard some important information for the learning process (46).

Oversampling (OS): this method balances the data by randomly resampling minority class instances. The weakness of this method is that if the dataset is large, it can introduce a significant additional computational load and the duplication of information due to the oversampling of the minority class instances, which can lead to the overfitting of the model. However, this method retains all important information, unlike the US method (44).

Hybrid sampling (HS): in this method, the dataset is balanced by combining the OS and US approaches (47).

## 5. Methods

Univariable BLR analysis was performed for all the independent variables. The odds ratios (ORs), the 95% confidence interval (CI), and the *p*-values were computed. The Spearman correlation coefficient between variables was calculated to minimize the redundancy between the variables. When the Spearman correlation coefficient was >0.6, the variable with the lowest significance value in the univariate BLR was selected. For the construction of the multivariate models, independent variables with a *p*-value < 0.2 in the univariate BLR models after the minimization of redundancies were considered and the oversample. For each of the non-linear CTGs variables with *p* < 0.2, univariate BLR models and Naive-Bayes models were developed. Asphyxia prediction models were implemented in R (48) using the Generalized Linear Models function to binomial logistic and the *naivebayes* package (49), respectively.

In constructing our models, we used a classification cut of 0.5. The evaluation of the model's fit was carried out by the analysis of the test of Hosmer and Lemeshow (50).

To allow an unbiased evaluation of the models, we use a two-fold cross-validation method: training and testing. The training dataset was randomly composed of 55% of the dataset and the test set for the remaining 45%. Due to the imbalance of the groups, asphyxia and no asphyxia, we used the technique of oversampling in the training dataset in order to have a probability of asphyxia of 0.3. Asphyxia prediction models were then built using resampling by oversampling the training set.

The oversampling technique was implemented using the *ROSE* R package (51). We repeated 1,000 times for each training set, obtaining BLR and NB models. We also calculated the area under the receiver operating characteristic curve (AUC) for the respective test set and their confidence interval.

A visual assessment of the histogram verified the normality of the distribution, and the median and interquartile interval were presented. In the case of categorical variables, we describe the absolute and relative frequency (in percentage).

## 6. Results

In Table 1, the data from the two groups used in this study are characterized. The APGAR score at 5^*th*^ minute >7 in 83% of the subjects in the non-asphyxia group, while for the asphyxia group is it verified in only 20% of cases. It is also worth mentioning that the APGAR score at 5^*th*^ minute was equal to 10 for 199 subjects in the non-asphyxia group and we do not have any case with this index in the asphyxia group. Also, the parturients in the asphyxia group had a higher BMI.

**Table 1:** Characteristics of asphyxia and non-asphyxia groups. Data are expressed in *n*(%) or median (interquartile interval).

**Variables**	**Non-asphyxia group**	**Asphyxia group**
	**(*n* = 502)**	**(*n* = 15)**
**Newborn**		
Birth weight (g)	3017.5 (2606.3–3377.5)	3215.0 (2982.5–3347.5)
Birth term (weeks)	39.1 (37.4–40.2)	39.3 (39.0–40.5)
Gender, male	288 (57%)	6 (40%)
Apgar >7 at 5 min	418 (83%)	3 (20%)
Artery pH	7.2 (7.1–7.3)	7.1 (7.0–7.2)
**Parturient**		
BMI (*kg*/*m*^2^)	24.3 (21.9–28.9)	28.8 (25.1–34.2)
Age (years)	32.0 (28.0–35.0)	30.0 (23.5–33.5)

BMI, body mass index.

**Figure 1:**
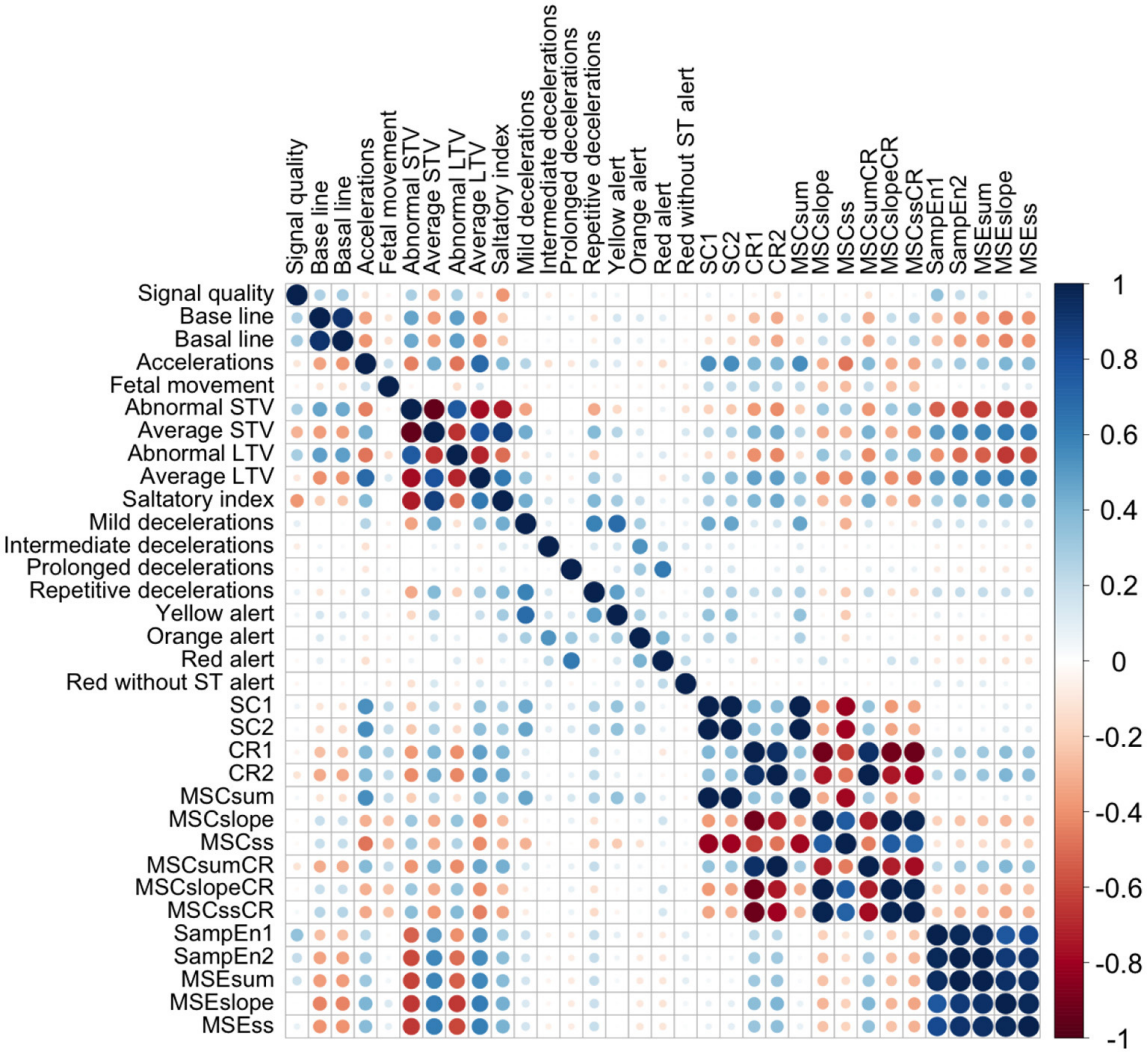
Correlation plot between CTGs indices. CR1, compression ratio for scale 1; CR2, compression ratio for scale 2; MSCslope, slope of the linear regression line best fitting the compressed file size all first five scales; MSCss, product of MSCsum and MSCslope; MSCsum, sum of the compressed file size values of the first five scales; MSCslopeCR, slope of the linear regression line best fitting the compression ratio values over on the first five scales; MSCssCR, product of MSCsumCR and MSCslopeCR; MSCsumCR, sum of the compression ratio values of the first five scales; MSEslope, slope of the linear regression line best fitting the entropy values over on the first five scales; MSEss, product of MSEsum and MSEslope; MSEsum, sum of the entropy values of the first five scales; SampEn1, entropy for scale 1; SampEn2, entropy for scale 2; SC1, compressed file size for scale 1; SC2, compressed file size for scale 2; STV, short-term variability; LTV, long-term variability.

The correlation between CTGs variables (linear and non-linear) can be seen in Figure 1. We obtained a high correlation within the entropy indices, and within some compression indices. We also obtained a high correlation between some SisPorto variables, such as: the average STV, abnormal STV, average LTV, abnormal LTV, and saltatory index. We obtained moderated correlation between the SisPorto variables and the non-linear indices, mainly between the entropy measures and the average STV, abnormal STV, average LTV, and abnormal LTV.

Univariable binary logistic regression analysis was performed for the 46 independent variables (13 clinical variables, 18 SisPorto variables, and 15 non-linear CTGs indices). The body mass index (BMI) variable presented 47 missings, and the missing values were replaced by the median BMI of mothers of the same age. To deepen our study, we considered independent variables with value *p* < 0.2 in univariate models (three clinical variables, five SisPorto variables, and five non-linear variables), and analyzed correlation between these variables to minimize the redundancy.

We found a coefficient >0.99 between the variables SC1, SC2, and MSCsum and between the variables CR2 and MSCsumCR. We also found a moderate/high correlation coefficient (*r* = 0.62) between the SisPorto variables prolonged deceleration and red alert. For the analysis, we used the variables with the lowest significance value for the outcome determined in the univariable BLR models (MSCsum, CR2, and prolonged deceleration). The remaining Spearman correlations were <0.6, so the remaining variables were used in constructing the models.

In Table 2, we present the characteristics of the asphyxia and non-asphyxia groups for independent variables with *p* < 0.2 after logistic regression analysis. Higher maternal age and male fetuses are significant protector factors against the risk of perinatal asphyxia. On the other hand, higher gestational age and a higher value for CTGs indices present significant risk factors for neonatal asphyxia. Furthermore, the increased number of accelerations and the presence of prolonged decelerations are also risk factors for neonatal asphyxia. The non-linear indices, CR2 and MSCsum, are the best separating individuals with asphyxia. Considering the entire dataset and defining for CR2 a threshold of 6%, we obtain a sensitivity of 87% (13 out of 15) and a specificity of 100%.

**Table 2:** Non adjusted odds ratio (OR), respective 95% confidence interval (CI), and *p*-value for variables with *p* < 0.2.

**Variables**	**Non-asphyxia**	**Asphyxia**	**OR**	**p-value**
	**(*n* = 502)**	**(*n* = 15)**	**[95% CI]**	
**Clinical**				
Maternal age (years)	32 (7)	30 (10)	0.92 [0.84;1.00]	0.054
Gestational age (weeks)	39.1 (2.8)	39.25 (1.45)	1.30 [1.00; 1.91]	0.117
Male fetus	288 (57%)	6 (40%)	0.50 [0.16; 1.39]	0.189
**SisPorto**				
Prolonged decelerations	41 (8%)	6 (40%)	7.50 [2.41;21.85]	<0.001
Accelerations	4 (8)	10 (12)	1.08 [1.01; 1.15]	0.014
Orange alerts	171 (34%)	9 (60%)	2.90 [1.03; 8.79]	0.046
Mild decelerations	6 (7)	8 (8.5)	1.09 [1.00; 1.18]	0.051
**Non-linear**				
CR2	4.32 (1.46)	8.78 (1.53)	9.79 [4.77; 30.54]	<0.001
MSCsum	1.46 (1.07)	2.60 (0.98)	4.55 [2.12; 10.90]	<0.001

Characteristics of asphyxia and non-asphyxia groups, expressed in *n*(%) or median (interquartile range). SisPorto and non-linear variables were obtained from CTGs in the last 60 min before birth. CR2, compression ratio scale 2; MSCsum, sum of compressed file size for all five scales.

For the construction of the multivariate logistic regression model, the clinical and SisPorto variables described in Table 2 were considered. As stated in methods section, the multivariate logistic regression model was performed using a oversampling technique in a two-fold cross-validation method. In Table 3, we the adjusted ORs and the respective 95% confidence interval for these models are presented. As a result, we only obtained three variables with *p*-value >0.05.

**Table 3:** Odds ratios obtained from the multivariate logistic regression model for the prediction of perinatal asphyxia based on clinical and SisPorto variables.

**Variables**	**OR [95% CI]**	***p*-value**
**Clinical**		
Maternal age	0.95 [0.90; 1.01]	0.126
Gestational age	1.72 [1.37; 2.24]	<0.001
Male fetus	0.35 [0.18; 0.63]	0.001
**SisPorto**		
Prolonged decelerations	63.18 [23.41; 190.25]	<0.001
Accelerations	1.11 [1.06; 1.18]	<0.001
Orange alerts	1.07 [0.54; 2.08]	0.848
Mild decelerations	1.02 [0.96; 1.08]	0.578

CI, confidence interval; OR, odds ratios.

In Table 4, we present the mean of the 1,000 values for AUC and respective 95% confidence interval (95% IC) for all asphyxia prediction models built. The Naive-Bayes model presented a higher AUC (92.55 [92.43; 92.68]) when considering clinical and SisPorto variables. However, the binary logistic regression presented better results (94.93 [94.55; 95.31]) using only the CR2 index.

**Table 4:** The area under the receiver operating characteristic curve and respective confidence interval of 95% obtained in the asphyxia prediction models.

**Variables**	**Binary logistic regression**	**Naive-Bayes**
	**AUC(%) [95% CI]**	**AUC(%) [95% CI]**
**Clinical and SisPorto**		
3 Clinical and 4 SisPorto	86.58 [86.41; 86.75]	92.55 [92.43; 92.68]
**Non-linear**		
CR2	94.93 [94.55; 95.31]	90.28 [89.80; 90.76]
MSCsum	79.60 [73.90; 80.09]	74.51 [73.90; 75.12]

AUC, area under the receiver operating characteristic curve; CR2, compression ratio scale 2. MSCsum, sum of compressed file size for all five scales.

## 7. Discussion

Our findings are notable for four main reasons. First, we used a retrospective database of CTGs along with clinical risk factors to predict perinatal asphyxia. Second, we use fHR data to predict perinatal asphyxia, allowing us to act before birth on detected cases. Third, the diagnosis is based on physiological signals having the advantage of being non-invasive and easily accessible. Fourth, a compression index seems to predict asphyxia.

The need for increasingly specialized health care and the early detection of pathologies justifies the importance of creating predictive models that aid diagnosis. BRL and Bayesian models have advantages and disadvantages, and their usefulness is remarkable when associated with traditional diagnostic methods. In Bayesian models, all relationships between variables are modeled. Therefore, it has an advantage over regression analysis due to its ability to capture the natural complexity of the data more effectively. BRL has the disadvantage of not being able to represent causality between the data. However, Bayesian models require the discretization of continuous data, which causes constraints and can reduce the model's performance. On the other hand, the notable advantages of Bayesian models are related to the fact that they can deal with missing data and allow a graphical representation that shows the relationship between the variables and their probabilistic values.

The incidence rate of perinatal asphyxia from the CHUSJ single birth data between 2010 and 2018 was 3.2 cases per 1,000, which agrees with the literature (3). Only 3,006 cases had pH analysis and CTGs recording in the last hour; of these only 15 were classified as having perinatal asphyxia. Only 21% of babies classified with asphyxia had CTG tracings in the last hour. However, the percentage of fetuses without pathology and CTG in the previous hour is similar. This fact leads us to consider that availableness of the CTG is arbitrary and that the lack of these data will be related to acquisition and storage problems.

The high correlation that we obtained between the entropy indices, the compression indices, and between the SisPorto indices did not surprise us. However, the correlation that we obtained between the linear and non-linear indices of the CTGs is surprising, and efforts should be made for the in-depth study of the relationships between these variables.

In this exploratory study, of the 13 clinical variables studied, only for three clinical variables (maternal age, gestational age, and fetal sex) we obtained a *p*-value < 0.2 when separating the groups. Clinical variables in the literature that were associated with the risk of asphyxia, such as third-trimester fetal weight percentile, fetal presentation, and increased labor with oxytocin, were not shown to be significant in our study. Moreover, clinical variables, such as prolonged rupture of membranes and multiple births, remained unanalyzed due to a need for more information in our database. The non-linear CTGs indices seem to be predictors of asphyxia, which showed a change in fHR variability in the 60 min before birth.

The multivariate models were built with the clinical and SisPorto variables described in Table 2 because the non-linear variables (CR2 and MSCsum) separate the study groups well. We built univariate asphyxia prediction models based on each of the non-linear variables to compare with the performance of the multivariate models. Previous studies suggested that the compression values depend on the data acquisition technique and machine. Two monitors were used in this dataset, the STAN, and the Philips Serie 50 Fetal Monitor, 20% of the pathological and 31% of the non-asphyxia CTGs were acquired from STAN. Also, 93% of the pathological and 68% of the non-asphyxia CTGs were ultrasound. The others were electrocardiograms.

The results of the models showed that the best model is the BRL univariate with CR2 variable. We obtained an AUC of 94.93 [94.55; 95.31], followed by the result obtained with the Naive-Bayes model using clinical and SisPorto variables (AUC of 92.55 [92.43; 92.68]). We recall that the multivariate models were built using oversampling techniques. A hold-one-out technique was also employed to substantiate the obtained results. For the CR2 variable, we obtained similar results with an AUC for the BRL model of 94.5 [85.3; 100.0] and the Naive-Bayes model of 89.1 [74.6; 100.0]. The results for the clinical and SisPorto variables were worse due to the absence of the oversampling technique in the training model and possibly due to some overfitting. We achieved an AUC for the BRL model of 77.2 [66.8; 87.7] and the Naive-Bayes model of 75.2 [63.9; 86.6]. These results reinforce the conclusion that the CR2 variable seems to discriminate fetuses with asphyxia from suspicious cases.

This study has some limitations, such as being a unicentric study, missing data, and being a retrospective study that might lead to different definitions of the diagnosis originating from the ICD-9-CM code.

Furthermore, the most critical limitations of this study are due to the reduced number of pathological cases and the imbalance between the number of subjects in the two groups. The latter will always be present in pathologies of low prevalence. An unbalanced dataset is a big challenge, as failure to address this issue can lead to biased classifiers. Undersampling can discard important information and, consequently, worsen the performance of some results. Oversampling tends to be the technique most used by researchers (46, 52).

Due to these limitations, before moving on to clinical practice, our results must be validated, in a future study, in a database with a larger number of pathological cases.

## 8. Conclusions

Correct and early detection of perinatal asphyxia enables adequate and specialized health care essential to reduce neonatal mortality. In recent decades, access to new clinical information has increased due to easier data acquisition and storage. The multiple systems allow combining several patient information not possible before.

The addition of vital and hemodynamic indicators, such as fHR analysis, in predicting perinatal asphyxia is critical. The integration of clinical parameters with linear and non-linear CTG indices leads to the developing of risk predictor models that are effective in early detection.

The model that best predicted perinatal asphyxia was the univariate binary logistic regression with the variable CR2 (AUC of 94.93 [94.55; 95.31]), demonstrating the importance of this variable in perinatal asphyxia detection.

Ideally, future studies should explore decision support systems to detect asphyxia, including clinical studies and CTGs resources (linear and non-linear).

## Data availability statement

The original contributions presented in the study are included in the article/supplementary material, further inquiries can be directed to the corresponding authors.

## Ethics statement

The Porto Retrospective Intrapartum Study was approved by the Centro Hospitalar Universitário de São João Ethics Committee in June 2018. The patients/participants provided their written informed consent to participate in this study.

## Author contributions

TH and CC-S conducted the conceptualization. MR and TH implemented and developed the method. MR, TH, and LC wrote and edited the manuscript. IN supervised all the clinical interpretations. All authors revised the paper critically for important intellectual content, made substantial contributions to the conception and design of the article, and agreed to the published version of the manuscript.
